# “More life and more days”—patient and care characteristics in a specialized acute pediatric palliative care inpatient unit

**DOI:** 10.1007/s00431-023-04813-8

**Published:** 2023-02-16

**Authors:** Sophie Stoesslein, Julia D. Gramm, Hans-Ulrich Bender, Petra Müller, Dorothee Rabenhorst, Gian Domenico Borasio, Monika Führer

**Affiliations:** 1 Center for Pediatric Palliative Care, Dr. von Hauner Children’s Hospital, University Hospital, LMU Munich, Marchioninistraße 15, Munich, 81377 Germany; 2 Palliative and Supportive Care Service, Lausanne University Hospital and University of Lausanne, Lausanne, Switzerland; 3 Pediatric Palliative Care, Department of Pediatrics, Bern University Hospital, Bern, Switzerland

**Keywords:** Specialized pediatric palliative care, Inpatient, Hospital units, Pediatrics, Palliative care

## Abstract

Only a few acute hospital inpatient units dedicated to pediatric palliative care (PPC) patients exist today. Clinical data on the patients and care provided at specialized acute PPC inpatient units (PPCUs) are scarce. This study aims at describing patient and care characteristics on our PPCU to learn about the complexity and relevance of inpatient PPC. A retrospective chart analysis was performed on the 8-bed PPCU of the Center for Pediatric Palliative Care of the Munich University Hospital, including demographic, clinical, and treatment characteristics (487 consecutive cases; 201 individual patients; 2016–2020). Data were analyzed descriptively; the chi-square test was used for comparisons. Patients’ age (1–35.5 years, median: 4.8 years) and length of stay (1–186 days, median 11 days) varied widely. Thirty-eight percent of patients were admitted repeatedly (range 2–20 times). Most patients suffered from neurological diseases (38%) or congenital abnormalities (34%); oncological diseases were rare (7%). Patients’ predominant acute symptoms were dyspnea (61%), pain (54%), and gastrointestinal symptoms (46%). Twenty percent of patients suffered from > 6 acute symptoms, 30% had respiratory support incl. invasive ventilation, 71% had a feeding tube, and 40% had full resuscitation code. In 78% of cases, patients were discharged home; 11% died on the unit.

*Conclusion*: This study shows the heterogeneity, high symptom burden, and medical complexity of the patients on the PPCU. The high dependency on life-sustaining medical technology points to the parallelism of life-prolonging and palliative treatments that is typical for PPC. Specialized PPCUs need to offer care at the intermediate care level in order to respond to the needs of patients and families.

**Table:** 

**What is Known:** *• Pediatric patients in outpatient PPC or hospices present with a variety of clinical syndromes and different levels of complexity and care intensity.* *• There are many children with life-limiting conditions (LLC) in hospitals, but specialized PPC hospital units for these patients are rare and poorly described.*	
**What is New:** *• Patients on a specialized PPC hospital unit show a high symptom burden and a high level of medical complexity, including dependency on medical technology and frequent full resuscitation code.﻿* *• The PPC unit is mainly a place for pain and symptom management as well as crisis intervention, and needs to be able to offer treatment at the intermediate care level.*

**What is Known:**

*• Pediatric patients in outpatient PPC or hospices present with a variety of clinical syndromes and different levels of complexity and care intensity.*

*• There are many children with life-limiting conditions (LLC) in hospitals, but specialized PPC hospital units for these patients are rare and poorly described.*

**What is New:**

*• Patients on a specialized PPC hospital unit show a high symptom burden and a high level of medical complexity, including dependency on medical technology and frequent full resuscitation code.﻿*

*• The PPC unit is mainly a place for pain and symptom management as well as crisis intervention, and needs to be able to offer treatment at the intermediate care level.*

## Introduction

The prevalence of life-limiting conditions (LLC) in children and adolescents is increasing [1–4]. In 2019, nearly 4000 children died from LLC in Germany [5]. Improvements in medical care and technology allow these patients to survive longer, albeit often without chances of cure. This leads to an increasing prevalence of children eligible for pediatric palliative care (PPC) and to longer duration of care [6, 7]. Internationally, various models and settings for PPC have been established: freestanding hospices, in-hospital PPC programs, and at-home PPC services [8–10]. These programs significantly differ in their structures and operations [11]. In the USA, hospital PPC programs focus on inpatient PPC consultation. In some countries, children’s hospices may include specialized PPC [9]. Acute inpatient PPC units are rare.

Despite the existing home PPC structures, many children with LLC are repeatedly hospitalized [3, 12]. Due to their complex diseases and care needs, they are often treated repeatedly on intensive care units (ICU) and frequently die there [13–16]. This raises the issue of appropriate care models for these patients and their diverse, complex care needs.

Our PPC center integrates a home PPC team and an 8-bed PPCU and offers in-hospital PPC consultations. The unit is specifically equipped to provide specialized acute inpatient PPC: interdisciplinary and multiprofessional staff with PPC training, technical equipment (e.g., for monitoring of ventilated patients and on-site EEGs), and family-friendly architecture (e.g., family space, private rooms). Patients may be admitted for pain and symptom management, stabilization in crises, or end-of-life care if the latter is not possible at home [6]. They receive medical treatment and nursing care, psychological and social support, spiritual care, and additional supportive therapies. Patients may be admitted to the PPCU even if life-prolonging treatment is still ongoing (integrated PPC).

The characteristics of PPC patients in home PPC, hospital-based consult services, hospice programs, or on general pediatric hospital units have been described [6, 9, 10, 12, 17–20]. However, data on specialized acute PPCUs are still scarce [21–25]. Few descriptions of comparable PPC programs exist, e.g., of inpatient hospice PPC in Canada [9]. A review of inpatient PPC programs in the USA showed a wide variability of services [11].

The aim of this study was to describe the patient population cared for on a specialized acute PPCU over more than 4 years in order to learn about the specificities, complexity, and relevance of inpatient PPC and to inform future PPC program planning. To our knowledge, this is the first systematic description of a large population of PPC inpatients in an acute specialized hospital setting.

## Materials and methods

### Setting

The study took place in the Center for Pediatric Palliative Care of the Munich University Hospital. The center comprises an 8-bed PPCU as well as a multiprofessional specialized home pediatric palliative care team which takes care of around 70–75 patients at any given time covering the region of Upper Bavaria, comprising urban and rural areas (17,500 km^2^; maximum distance from the center approx. 160 km). The home PPC team provides 24/7 coverage and allows up to 84% of patients to die at home. Its activity is described in detail in ref. [6].

The center is part of the university’s children’s hospital. It cares for children and adolescents suffering from life-limiting conditions (LLC), from the prenatal stage up to usually 18 years of age. In Germany, patients older than 18 years can remain under pediatric treatment if their disease and their physical and cognitive impairments make this the better choice. Therefore, the center also cares for individual adult patients that are up to 35 years old, severely impaired, and with a LLC originating in childhood.

Most children taken care of on the PPCU are referred by the home PPC team, usually due to a crisis that cannot be adequately managed in the home setting (symptom control and /or psychosocial issues). The team of the PPCU consists of pediatricians, nurses, social workers, psychologists, spiritual assistants, and special need educators with a specific palliative care training. The goal of care is stabilizing the situation and allowing the return home as soon as possible.

### Sampling

We carried out a single-center, retrospective chart review of 487 consecutive cases on a specialized acute PPCU. The 487 cases relate to 201 individual patients treated on the PPCU between April 2016 and November 2020. Every admission represents one case. All cases were included in the analysis.

### Data collection

Patient data were retrieved from the patient charts and discharge letters. Data were anonymized directly during data retrieval. The following variables were extracted: (1) patient characteristics and clinical context (gender, age, main diagnosis, symptoms, referring institution, length of stay, types of discharge, resuscitation status) and (2) diagnostic procedures, medical treatments (diagnostic imaging, therapeutic interventions, technical devices, medication), and intensity of psychosocial care.

Symptoms were differentiated into permanent, controlled symptoms (“permanent symptoms”), and non-controlled symptoms requiring therapeutic interventions (“acute symptoms”). The drugs administered as long-term medication were extracted from the medication plans at discharge for the consecutive cases of 1 year only (1–12/2020, *n* = 102) due to the large volume of data. The intensity of psychosocial care provided to patients and their relatives was rated retrospectively by the treating psychosocial professionals (psychologists, pastoral workers, social workers) for all cases within 1 year (1–12/2020, *n* = 102), based on their documentation and knowledge of the cases. The intensity was scored on a 4-point scale: 1 = no contact, 2 = only one initial consultation, 3 = several accompanying consultations, and 4 = intensive support and care (i.e., regular, therapeutic interventions with patient/family members). The patients’ main diagnoses were assigned to groups according to Together for Short Lives (TfSL, Table 1) [26] and the International Statistical Classification of Diseases and Related Health Problems, 10th Revision (ICD-10) [27].

**Table 1:** TfSL categories of life-limiting and life-threatening conditions [26]

**TfSL group 1**	Life-threatening conditions for which curative treatment may be feasible but can fail
**TfSL group 2**	Incurable conditions, where premature death is inevitable, but long periods of intensive disease-directed treatments aim at prolonging life and taking part in normal activities
**TfSL group 3**	Progressive conditions without curative treatment options; treatment is exclusively palliative and may commonly extend over many years
**TfSL group 4**	Irreversible but non-progressive conditions that cause severe disability, with a high likelihood of health complications and premature death; palliative care may be required at any stage, with unpredictable crises and intermittent episodes of care

*TfSL* Together for Short Lives

The study protocol was reviewed and approved by the Munich University Hospital Ethics Committee in December 2020 (project no.: 19–2519).

### Data analysis

Data were analyzed descriptively and case-based (i.e., referring to *n* = 487 consecutive cases) in order to depict a realistic image of the actual care provided on the PPCU. Only statistical comparisons of disease groups (TfSL, ICD-10) were performed referring to *n* = 201 individual patients, since the main underlying diseases are stable patient features, and a case-based analysis would lead to an overrepresentation of repeatedly admitted patients. The chi-square test was performed for group comparisons, using the Bonferroni correction. Numeric variable distributions are described by median, range, and interquartile range (IQR) because of skewness. Missing data were recorded and reported in the descriptive statistics. Thus, some reported frequencies may not add up to 100%. Most statistics are based on complete data. For the chi-square tests for group comparisons, there were no missing data, and no imputation or deletion technique was required. For statistical analysis, SPSS Software Version 26 was used (SPSS, Inc. Chicago Illinois).

## Results

### Patient, admission, and discharge characteristics

Table 2 shows patient and care characteristics for the 487 consecutive cases (= 201 individual patients). The patients’ age showed a wide range from neonates to young adults (median: 4.8 years, range: 0–35.3, IQR: 1.4–12.5). In 19% of cases (*n* = 92/487), the patient was an infant (≤ 1 year; with *n* = 14/92 neonates), and in 12% over 18 years old (*n* = 58/487). In 46% of cases, patients had a migration background (i.e., either the patient or their parents had migrated to Germany), and in 18% of cases, an interpreter was needed due to language barriers. In 37% of cases, the patient was admitted without an accompanying adult.

**Table 2:** Patient and care characteristics of 487 cases on the PPCU, 04/2016–11/2020

*All cases n* = *487*	*n (%)*	*Median (IQR)*	*Range*
*Gender*			
*Male*	297 (61)		
*Female*	190 (39)		
*Age (years)*		4.8 (1.4–12.5)	0–35.3
*0–1*	92 (19)		
> *1–4*	139 (29)		
> *4–14*	152 (31)		
> *14–18*	46 (9)		
> *18*	58 (12)		
*Length of stay (days)*		11 (6–21)	1–186
*Referring institution*			
*Home PPC team*	322 (66)		
*ICU*	46 (9)		
*Other hospital units*	119 (25)		
*Discharge*			
*Home*	377 (77)		
*Transferred to other unit/hospital*	56 (12)		
*Deceased on PPCU*	54 (11)		
*Full CPR code*	196 (40)		

*ICU* intensive care unit, *PPC* pediatric palliative care, *IQR* interquartile range

#### Admissions and discharges

Admission and discharge information are listed in Table 2. Additionally, Table 3 shows TfSL group comparisons for admission frequency and deaths on the PPCU. Sixty-six percent of cases on the PPCU were referred by a home PPC team and 9% by an ICU (Table 2). More than 1/3 of individual patients were admitted repeatedly (38%, *n* = 77/201 patients; median: 3, range: 2–20, IQR: 2–6 admissions for patients with > 1 admission). Patients in TfSL group 1 were more often admitted only once (81%; and 92% of oncological patients). Repeated admissions were more often in TfSL group 4 (47%; *χ*^2^ (3) = 9.277, *p* = 0.026; Table 3). The length of stay varied greatly from 1 to 186 days, but most were under 3 weeks (median: 11 days, IQR: 6–21; Table 2). In 78% of cases, patients were discharged home.

**Table 3:** Comparison of TfSL groups: number of admissions and deaths per group

	***Total***	***TfSL group 1***	***TfSL group 2***	***TfSL group 3***	***TfSL group 4***	*p*
*n (% of 201)*	201 (100)	41 (20)	19 (9)	53 (26)	88 (44)	
***Repeated admissions***						*p* = 0.026
> *1 admission*	77 (38)	8 (19.5)	6 (32)	22 (41.5)	41 (47)	Group 1 vs group 4
***Deceased on PPCU***						*p* < 0.001
*Yes*	54 (27)	23 (56)	2 (10.5)	14 (26)	15 (17)	Group 1 vs all other groups

The chi-square test with *n* = 201 individual patients. Pairwise comparison of column proportions using the Bonferroni correction

*TfSL* Together for Short Lives, *PPC* pediatric palliative care

In 11% of cases, the patient died on the PPCU (54/487; 27% of individual patients, *n* = 54/201). The length of end-of-life stays also varied considerably (median: 8 days, range: 1–101, IQR: 4–20). Of the 54 patients who died on the PPCU, 50% were < 3.5 years (IQR: 0.6–13), and 35% were infants < 1 year. Comparing the TfSL groups, significantly more patients died on the PPCU in TfSL group 1 (56%) than in the other groups (*p* < 0.001; Table 3). Oncological patients comprised 59% of TfSL group 1 patients (*n* = 24/41); most oncological patients died on the PPCU (79% vs. 21% of non-oncological patients, *n* = 19/24 vs. *n* = 35/177; *p* < 0.001).

The patient’s resuscitation code status was retrievable from the digitalized patient chart in 73% of cases (*n* = 355/487). In 40%, full cardiopulmonary resuscitation (CPR) was requested (*n* = 196/487; 55% of the 355 cases with documented code). Among the 77 (38% of 201) repeatedly admitted patients, 63 (82% of 77) had no change in their code status over time, 9 (12%) changed to no CPR, 2 (3%) changed from no to full CPR, and 3 (4%) had repeated status changes. An advance directive (AD), mostly of the parental type [for details, see ref. 28], was available in 21% of cases (*n* = 100) and in 19% of deceased patients.

### Diseases and clinical symptoms

**Fig. 1:**
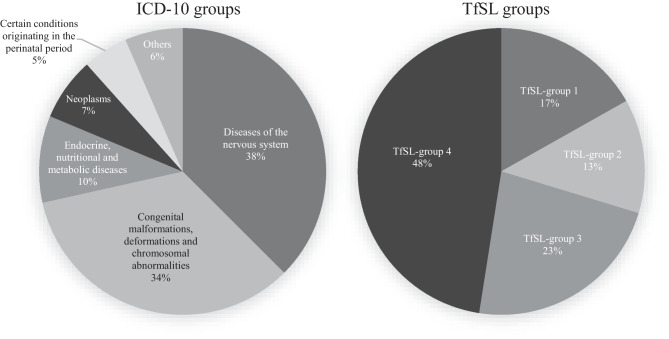
Underlying diseases by ICD-10 disease groups and TfSL groups, *n* = 487 cases, 04/2016–11/2020. ICD-10, International Statistical Classification of Diseases and Related Health Problems, 10th revision; TfSL, Together for Short Lives. Category “Others” = non-frequent conditions grouped together, e.g., injuries, poisoning, circulatory system diseases, and diseases not otherwise classified

Regarding the underlying diseases, there were 105 different diagnoses out of 11 different ICD-10 disease groups. Figure 1 displays the proportions of ICD-10 and TfSL groups for all cases. Main diagnostic groups were diseases of the nervous system (38%) and congenital abnormalities (34%). Oncological diseases comprised only 7% of cases, but 12% of individual patients due to the paucity of readmissions in this group.

In most cases, patients suffered from multiple severe symptoms. The most frequent permanent symptoms were neurological symptoms (88% of cases, *n* = 429/487, of which 78% had seizures, 47% spasticity, 21% dystonia). Permanent gastrointestinal symptoms, mainly constipation, were also very frequent (80% of cases).

**Fig. 2:**
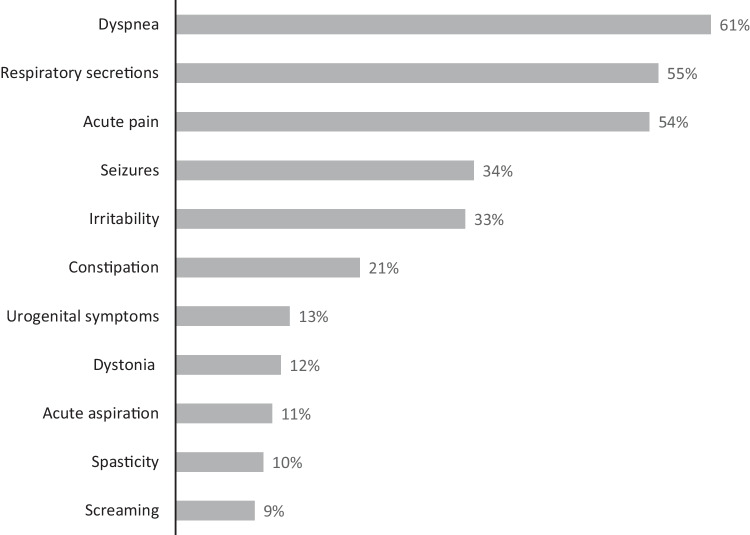
Acute clinical symptoms requiring treatment initiation/adjustment during the stay on the PPCU, *n* = 487 cases, 04/2016–11/2020

Figure 2 shows the frequencies of all registered acute symptoms. The most frequent acute symptoms were dyspnea (61%), pain (54%), and gastrointestinal symptoms (46%, *n* = 224/487, of which 59% had vomiting episodes, 47% constipation, 23% diarrhea,). In 58% of cases, patients suffered from more than two acute symptoms, in 20% from at least six acute symptoms. For the subset of cases with oncological diseases (7%, *n* = 36/487), the frequencies of acute symptoms were acute pain (89%), constipation (53%), dyspnea (39%), irritability (28%), urogenital symptoms (17%), respiratory secretions (14%), seizures (11%), acute aspiration (8%), spasticity (6%), dystonia (3%), and screaming (0%).

### Diagnostics and treatment

#### Diagnostics and interdisciplinary consultations

In 47% of cases (*n* = 234/487), technical diagnostic procedures were performed, including magnetic resonance imaging (MRI), computer tomography (CT scan), X-ray imaging, ultrasound, electroencephalogram (EEG), and electrocardiogram (ECG). In 18% of these cases, the diagnostics were performed under sedation (*n* = 43/234). In 15% of all cases, other pediatric subspecialties were consulted (e.g., orthopedics, neurology, ophthalmology). In 13 cases (3%), the clinical ethics committee was consulted for complex decision-making, e.g., withdrawal of artificial nutrition and hydration.

#### Medical technology and medication

In 80% of cases, patients were dependent on medical technology. Table 4 shows the medical devices and parenteral medication in the 487 consecutive cases. Thirty percent of patients had respiratory support, 5% had invasive, and 11% had non-invasive ventilation. No intubations were performed on the PPCU, while in some cases, non-invasive ventilation was initiated on the unit. Seventy-five percent of patients suffered from dysphagia (*n* = 363/487), and in most of these cases, they had some type of feeding tube (85%, *n* = 307/363).

**Table 4:** Medical devices and parenteral medication of 487 cases, 04/2016–11/2020

	*n (%)*
***Medical devices***	
* Respiratory support*	145 (30)
* Non-invasive ventilation*	54 (11)
* Invasive ventilation*	23 (5)
* Tracheostomy without ventilation*	33 (7)
* High-flow therapy*	35 (7)
* Feeding tube*	347 (71)
* PEG only*	199 (41)
* PEG plus PEJ*	30 (6)
* PEJ*	48 (10)
* Nasogastric tube*	70 (14)
***Parenteral medication***	
* Intravenous*	224 (46)
* Subcutaneous*	18 (4)

*PEG* percutaneous endoscopic gastrostomy, *PEJ* percutaneous endoscopic jejunostomy

Parenteral medication (most often antibiotics) was administered in 50% of cases, mostly intravenously (93% of parenteral medication cases, *n* = 224/242). We analyzed the drugs administered as long-term medication in all consecutive cases over 1 year (1–12/2020, *n* = 102). The most common drugs were antacids, laxatives, and anticonvulsants (in 69%, 65%, and 61% of cases, respectively). The average number of different drugs per day per case was 10 (mean/median:10, range: 0–20, IQR: 7–13). In 50% of cases, patients received inhalation therapy with beta-mimetics, corticosteroids, and/or ipratropium bromide (frequency median: 4 times/day, range: 1–8, IQR: 3–4).

#### Psychosocial care

In 88% of 102 cases within 1 year (1–12/2020), there was a contact between the patient/family and the psychosocial/spiritual team. In most cases, the psychosocial care was rated as intensive (68%, *n* = 69/102), including regular contact and therapeutic interventions with the whole family system and/or individual family members.

## Discussion

This study describes the heterogeneity and care complexity of PPC patients treated at our specialized PPC unit over a 4-year-period. Our patients were mostly neurologically impaired, had multiple severe symptoms, and received complex non-pharmacological and pharmacological treatment. The vast majority was dependent on life-sustaining measures, mostly artificial nutrition and/or ventilatory support. Patients were rarely admitted for end-of-life care, which is explained by the fact that 84% of the patients cared for by our home PPC team are able to die at home [6]. Since the home PPC team and the unit are located in the same center, and clinical collaborators rotate between the two, this allows for maintaining continuity of care over a period of often months to years [6].

Most PPCU admissions took place in crisis situations to achieve symptom control and stabilization. The most prevalent acute symptoms were dyspnea, respiratory secretions, and pain. Similar levels of medical complexity and symptom burden have been described for other inpatient PPC services (inpatient hospice, hospital PPC consults) [9, 17].

Our data show a considerable variation in care needs, admission, and discharge patterns between disease groups. Among the ICD-10 disease groups, neurological and congenital conditions had the highest prevalence. This distribution of clinical conditions is similar to that in our home PPC team and in children with LLC in hospitals [3, 6, 17, 18]. The small number of TfSL-2 patients (e.g., cystic fibrosis, Duchenne muscular dystrophy) in the cohort may be explained by new treatment options for these patients that allow them to survive into adulthood [29].

In accordance with the literature, most of our patients presented with non-oncological diseases [6, 18, 20, 30], which is in stark contrast to adult palliative care [19, 31, 32]. Most of the TfSL-1 patients, particularly oncological patients, had one single admission to our PPCU, often for end-of-life care. In an analysis of an inpatient PPC hospice in Canada, cancer patients also had the lowest number of admissions [9]. This is likely due to rare and late referrals, coupled with rapid disease progression in this group [6]. The TfSL-1 diseases are often associated with long periods of cure-oriented therapy and high hospital use [33]. In our clinical experience, the advent of last-generation treatments such as immunotherapies appears to contribute to the rarity and lateness of PPC referrals for these patients, many of whom currently end up dying in ICUs [34, 35].

Our data indicate that some PPC patients, particularly in the TfSL groups 3 and 4, may repeatedly need hospital treatment in crises, despite receiving specialized PPC at home. This confirms data showing a high hospital use and medical complexity of children with chronic complex and life-limiting conditions [7, 12, 15, 33, 36, 37]. Studies report higher care needs and longer hospital stays, as well as a high percentage of ICU admissions and deaths for these patients [13–16]. In accordance with these data and our clinical experience, many patients eligible for PPC are currently treated in pediatric ICUs. Child stays and deaths in ICUs are known to be stressful and associated with a high risk for complicated grief of parents [38–40]. A specialized PPCU appears to offer an alternative better suited to the needs of patients with LLC requiring intensive medical treatment focused on symptom control that cannot be delivered in the home setting.

The care level on our PPCU is comparable to a pediatric intermediate care unit. Such units have been established in hospitals to spare ICU resources, by offering intensive care and monitoring for less critically ill patients who still require a higher care level than general hospital care [41, 42]. The PPCU differs from an intermediary care unit in that only children with an LLC are accepted, and a special emphasis is placed on psychological, social, and spiritual care, as well as advance care planning. The focus is the quality of life of the child and the entire family. This does not mean forsaking all life-prolonging measures: in pediatrics, hope for life-prolongation and palliative care often go hand in hand over long periods of time. as opposed to adult palliative care [19]. Of note, in two-thirds of cases on our PPCU, patients had either full code status or no documented limitation of life-sustaining measures. This is in keeping with the stated intent of PPC to not be limited to end-of-life care, but to strive for early integration in the patients’ treatment, which may still include life-prolonging therapies [43, 44]. Patients and families may adjust their treatment goals and preferences in the course of their disease, which is supported by our data (18% of repeatedly admitted patients had changes in CPR status).

Decisions about limitations of life-sustaining treatments in children with LLC are challenging and fraught with a high emotional burden, making it important to clarify goals of care well in advance of the terminal phase [45–48]. Pediatric advance care planning (ACP) allows families to prepare for future crises and plan the end-of-life care [45–47, 49, 50], but it often takes place too late in the course of the illness [51–53]. We have found the PPCU to be a very good place to initiate ACP discussions that can then be continued in the home care setting using a structured approach ﻿[28].

Hospital PPC consult teams have been promoted as a way to deliver bedside PPC in all inpatient units including ICUs. However, a recent analysis of such inpatient PPC programs in the USA revealed problems of practice quality and staff shortage [11]. Other efforts focus on guidelines and staff training for ICUs to incorporate PPC into their practice [54, 55]. However, even if it is essential for ICU clinicians and nurses to acquire basic PPC skills, ICUs generally lack the specialized PPC staff and the appropriate environment required for family-centered and comprehensive PPC [56]. In this respect, the high intensity of psychosocial care observed on our PPCU is consistent with recent data reporting a high burden of care and distress in parents of children with LLC, as well as unmet psychological and practical support needs [57, 58].

Our PPC center comprises a PPCU, a home PPC team, and a hospital PPC consult team. This helps to integrate PPC in existent hospital structures, facilitates continuity of care, and supports early integration of PPC for children with LLC. PPCUs are very resource-intensive and probably best situated in PPC centers at tertiary pediatric hospitals, from where they can cooperate with all regional healthcare providers involved in the care of children with LLC.

### Limitations

One main limitation of our study is the retrospective design, which led to some missing or incomplete data. In addition, not all clinical features of interest could be reliably retrieved from the medical charts. Since we used a case-based approach, repeatedly admitted patients may be overrepresented in the sample. And due to the monocentric design, the generalizability of the results to other settings or care services may be limited.

## Conclusion

Our study describes the patient population and care offered on a specialized PPCU. The care level required to adequately respond to the needs of the patients and families corresponds to that of an intermediate care unit. Consistent with the parallelism of life-prolonging and palliative treatments in PPC patients, most patients on our PPCU did not have limitations on life-sustaining treatments and showed a high medical complexity and dependency on medical technology. An acute PPCU mainly performs symptom management and crisis intervention, including psychosocial crises. Most patients are discharged home, with end-of-life care taking place only in a minority of cases.

Our data may inform further PPC program development and stimulate PPC network building. Prospective studies should further investigate admission reasons, features, and outcomes of the medical, nursing, psychosocial, and spiritual care on a PPCU for patients with LLC, in comparison with other pediatric inpatient units. Such data could strengthen the rationale for the establishment of a limited number of acute PPCUs to complement outpatient PPC care and hospital PPC consult teams in selected tertiary centers.

## Abbreviations

ACP: Advance care planning
AD: Advance directive
CPR: Cardiopulmonary resuscitation
CT: Computer tomography
EEG: Electroencephalograms
ECG: Electrocardiograms
ICD-10: International Statistical Classification of Diseases and Related Health Problems, 10th Revision
ICU: Intensive care unit
IQR: Interquartile range
LLC: Life-limiting conditions
MRI: Magnetic resonance imaging
PPC: Pediatric palliative care
PPCU: Pediatric palliative care inpatient unit
TfSL: Together for Short Lives
